# Characterization of cerebral macro- and microvascular hemodynamics during transient hypotension

**DOI:** 10.1152/japplphysiol.00743.2022

**Published:** 2023-08-10

**Authors:** Leena N. Shoemaker, Daniel Milej, Aleena Sajid, Jigneshkumar Mistry, Keith St Lawrence, J. Kevin Shoemaker

**Affiliations:** ^1^Imaging Program, Lawson Health Research Institute, London, Ontario, Canada; ^2^Department of Medical Biophysics, Western University, London, Ontario, Canada; ^3^School of Kinesiology, Western University, London, Ontario, Canada; ^4^Department of Physiology and Pharmacology, Western University, London, Ontario, Canada

**Keywords:** cerebral blood flow, cerebrovascular, diffuse correlation spectroscopy, lower body negative pressure, transcranial Doppler

## Abstract

The aim of the current study was to establish the interplay between blood flow patterns within a large cerebral artery and a downstream microvascular segment under conditions of transiently reduced mean arterial pressure (MAP). We report data from nine young, healthy participants (5 women; 26 ± 4 yr) acquired during a 15-s bout of sudden-onset lower body negative pressure (LBNP; −80 mmHg). Simultaneous changes in microvascular cerebral blood flow (CBF) and middle cerebral artery blood velocity (MCAv_mean_) were captured using diffuse correlation spectroscopy (DCS) and transcranial Doppler ultrasound (TCD), respectively. Brachial blood pressure (finger photoplethysmography) and TCD waveforms were extracted at baseline and during the nadir blood pressure (BP) response to LBNP and analyzed using a modified Windkessel model to calculate indices of cerebrovascular resistance (Ri) and compliance (Ci). Compared with baseline, rapid-onset LBNP decreased MAP by 22 ± 16% and Ri by 14 ± 10% (both *P* ≤ 0.03). Ci increased (322 ± 298%; *P* < 0.01) but MCAv_mean_ (−8 ± 16%; *P* = 0.09) and CBF (−2 ± 3%; *P* = 0.29) were preserved. The results provide evidence that changes in both vascular resistance and compliance preserve CBF, as indexed by no significant changes in MCAv_mean_ or DCS microvascular flow, during transient hypotension.

**NEW & NOTEWORTHY** To characterize the relationship between cerebrovascular patterns within the large middle cerebral artery (MCA) and a downstream microvascular segment, we used a novel combination of transcranial Doppler ultrasound of the MCA and optical monitoring of a downstream microvascular segment, respectively, under conditions of transiently reduced mean arterial pressure (i.e., lower body negative pressure, −80 mmHg). A rapid increase in vessel compliance accompanied the maintenance of MCA blood velocity and downstream microvascular flow.

## Graphical abstract

**Figure:**
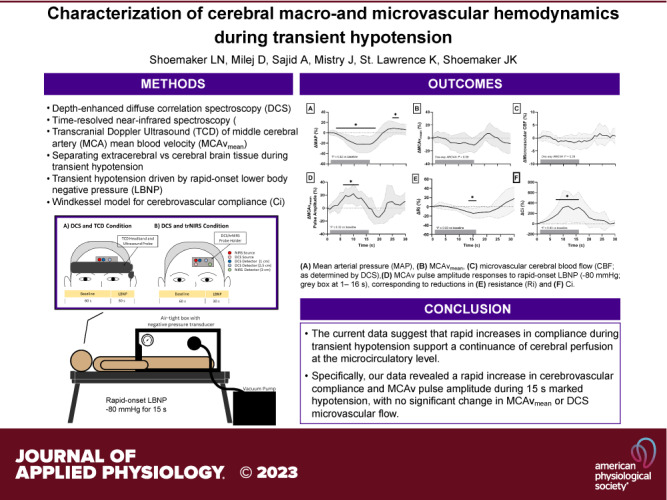

## INTRODUCTION

Stability of cerebral blood flow across a range of perfusion pressures is achieved by adjustments in vasoactive properties in both the large arteries at the base of the brain (1, 2) and the small microvessels that perfuse the parenchyma to support neuronal metabolism (3). The primary vasoactive responses include changes to vascular stiffness and resistance achieved through a combiation of myogenic, metabolic, and endothelial shear mechanisms. The range of perfusion pressures over which this autoregulatory feature of cerebrovascular control is debated (4) but the fundamental premise suggests that blood flow in the large and smaller diameter vascular segments should be consistent with each other.

The ability to quantify dynamic flow patterns along the conduit-to-microcirculation segments of a cerebral vascular bed is limited in the conscious human due to the confines of the rigid skull (5–8). In many cases, measures made at the conduit artery segment are interpreted to include downstream events (i.e., microcirculation), although distinction of blood flow patterns in these two vascular segments cannot be determined without concurrent and direct analysis of the microvasculature. In this regard, little is known about the microcirculation and its ability to respond rapidly to changes in perfusion pressure.

Recent advances in optical technologies, specifically the development of diffuse correlation spectroscopy (DCS) (9), make it possible to measure cerebral blood flow (CBF) noninvasively after accounting for extracranial perfusion. The combination of DCS with transcranial Doppler ultrasound (TCD) enables simultaneous measurements of the hemodynamic nature of CBF in cerebral arterioles/capillaries and large cerebral artery vascular beds (6), respectively. Considering the high temporal resolution of both technologies, their combination enables a greater understanding of concurrent cerebrovascular changes during highly dynamic and real-life scenarios. This is a novel approach and only a handful of studies have explored both cerebral macro- and microvascular function in adult humans. Available studies report good agreement between macro- and microvascular vasomotor states at rest in clinical (6) and healthy (7) populations, as well as during tests of dynamic cerebral autoregulation (8) and cerebrovascular reactivity to acetazolamide (5). However, the relationship between large cerebral artery and microvascular patterns of control during rapid and transient changes in blood pressure remains poorly understood.

In addition to the expected but unmeasured continuity of dynamic flow patterns across the cerebrovascular bed, the potential mechanisms that underly this regulation remain incompletely understood, particularly in rapidly changing pressure. Whereas changes in vascular resistance (Ri) form the principle concept of control, recent modeling advances and experimental measures implicate dynamic changes in arterial compliance as well (10) that, when measured directly, precede active changes in Ri. The purpose of this rapid change in vascular compliance (Ci) may be speculated to preserve downstream perfusion (i.e., perfusion occurring beyond the MCA and toward the microvasculature) during the period of transient hypotension before the autoregulatory vasodilatory response.

Our primary objective in this study was to establish the relationship between blood flow in the large middle cerebral artery (MCA), reflecting patterns affected by the entire vascular bed, and the corresponding flow in the downstream microvasculature under conditions of transiently reduced blood pressure. In addition, the potential roles played by Ri and Ci in these patterns were assessed. To complete this aim, we utilized simultaneous depth-enhanced DCS with TCD during a 15-s bout of rapid-onset lower body negative pressure (LBNP). We further combined the DCS measurements with depth-enhanced time-resolved near-infrared spectroscopy (trNIRS) to quantify changes in tissue oxygen saturation and cerebral blood volume. We tested the hypothesis that increases in Ci and reductions Ri in response to hypotension, would underly the maintenance of cerebral vascular bed perfusion and oxygenation.

## METHODS

All procedures were approved by the Health Sciences Research Ethics Board (HSREB) at Western University (No. 112633) and Lawson Health Research Institute (No. 107985), adhering to the guidelines of the Tri-Council Policy Statement for research involving humans as well as those set forth in the Helsinki Declaration. Participants provided written informed consent following verbal and written explanation of the experimental procedures.

Ten healthy participants (5 women, 26 ± 4 yr, 177 ± 6 cm, 75 ± 15 kg) were recruited. Before tests, participants completed a mandatory health history form to evaluate the inclusion/exclusion criteria. Participants were included if they reported being a nonsmoker with no prior diagnosis of cardiovascular disease, neurological disorder, diabetes, or hypertension. All subjects reported that they identify with their sex assigned at birth. One male participant was excluded as a result of insonation difficulties of the MCA from a poor temporal window. Therefore, a total of nine participants (*n* = 9) were subsequently included in the analysis. To control for potential influences of sex hormones on vascular function, female participants were tested during the luteal stage of menstruation (18 ± 2 day). Furthermore, participants reported to the Laboratory for Brain and Heart Health at Western University for testing following a 4-h fast (including caffeine) and 12 h of abstinence from alcohol consumption and physical activity.

### Experimental Protocol

**Figure 1.**
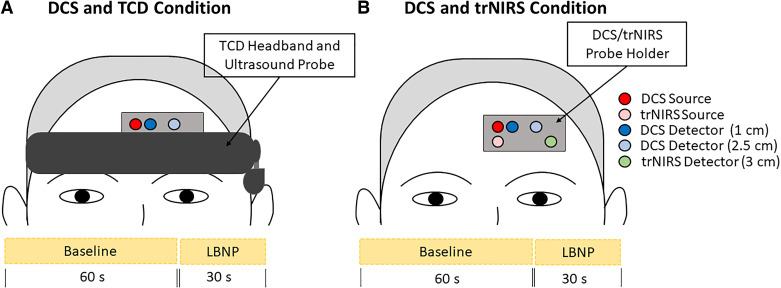
Illustration of the optical probe and transcranial Doppler (TCD) ultrasound placement for the diffuse correlation spectroscopy (DCS) and TCD condition (*A*) and the DCS and time-resolved near-infrared spectroscopy (trNIRS) condition (*B*). Light sources and detectors on the probe holder are color-coded. Red, DCS source; pink, trNIRS source; dark blue, DCS detector (*r*_SD_ = 1 cm); light blue, DCS detector (*r*_SD_ = 2.5 cm); green, trNIRS detector (*r*_SD_ = 3 cm). Experimental paradigms used in the study are illustrated as a 60 s baseline followed by 15 s bout of lower body negative pressure (LBNP) and 15 s recovery.

The primary purpose of this study was to assess the coordination of macrovascular and microvascular perfusion of the brain during a hypotensive episode. The secondary purpose was to study the patterns of change in cerebrovascular resistance (Ri) and compliance (Ci) that support the macro-microvascular coordination. Our primary outcomes required the use of optical monitoring equipment (i.e., DCS and trNIRS) to measure microvascular hemodynamics and TCD to measure MCA and downstream microvascular hemodynamics. Due to limited space on the head, it was not possible to instrument the participants with trNIRS, DCS, and TCD simultaneously. Therefore, two protocols were performed in a randomized order (computer-generated allocation): a TCD-DCS condition and a trNIRS condition. The latter was used to measure microvascular tissue oxygen saturation (StO_2_) and total hemoglobin (HbT), which is a marker of relative cerebral blood volume. Figure 1 illustrates the placement of probes on the participant’s head for both the TCD-DCS and trNIRS conditions.

To create sustained but brief (15 s) bouts of orthostatic stress, participants were sealed from the waist down in an LBNP chamber. LBNP is a commonly used technique to mimic orthostatic stress while the participant remains in a supine posture. This technique is advantageous for the current study design, as it enables control over the intensity and timing of each bout of hypotension while minimizing movement of the participant. The latter is necessary for the highest quality data collection with optical monitoring technologies. Both the TCD-DCS and trNIRS conditions included one short (15 s) pulse of rapid-onset LBNP at −30 mmHg and another at −80 mmHg, followed by a 15-s recovery (Fig. 1). To avoid crossover effects, there was >10 min between the TCD-DCS and trNIRS conditions. The suction intensities were randomized (computer-generated allocation) with at least 2 min of recovery between them. The lower LBNP intensity (i.e., −30 mmHg) was used to produce similar sensory effects to −80 mmHg while having a minor effect on blood pressure.

### Instrumentation

#### Systemic variables.

While supine, participants were instrumented with a standard electrocardiogram (ECG; ADInstruments Bio Amp FE132, Bella Vista, New South Wales, Australia), from which continuous heart rate (HR) was calculated. Finger photoplethysmography (Finometer model 1, Finapres Medical Systems, Enschede, The Netherlands) provided continuous arterial blood pressure measurements at the finger that was calibrated against three manual sphygmomanometric brachial artery measures, and the 1-s delay in the analog output was accounted for before data analysis. Expired gas was sampled at the mouth for the measurement of partial pressure end-tidal CO_2_ ($\text{P}{\text{ET}}_{{\text{CO}}_{2}}$; ML206 analyzer).

#### Perceived Feeling Scale.

To account for any psychosomatic influences on cardiovascular function, participants were asked to report their “feeling” (overall feeling state) at baseline and immediately after each bout of rapid-onset LBNP, using an 11-point affect feeling scale.

#### Transcranial Doppler.

Cerebral blood velocity was measured as the instantaneous peak velocity in the right MCA (M1 segment) using TCD (Neurovision TOC2M, Multigon Industries, Elmsford, CA). The 2 MHz frequency ultrasound probe was placed on the transtemporal acoustic window and set to a depth of 47–55 mm. Ipsilateral carotid compression was used to ensure insonation of the MCA in each participant. The probe was then secured in place with a headband device to maintain insonation angle and position.

Doppler ultrasound and systemic variables were sampled at 1 kHz using a data acquisition system (PowerLab 16/35SP, ADInstruments) and software (LabChart 8, ADInstruments) and stored for subsequent offline analysis. MCA pulse amplitude was calculated as the difference of each cardiac cycle’s MCA systolic peak and diastolic nadir. MCA pulsatility index was calculated as pulse amplitude divided by MCAv_mean_.

#### Diffuse correlative spectroscopy.

Diffuse correlation spectroscopy is an optical technique that uses near-infrared light to noninvasively measure microvascular flow (9). Unlike NIRS, which measures light absorption, DCS measures light intensity fluctuations caused by the movement of light scatterers (11). In tissue, this phenomenon is dominated by the flow of red blood cells through the microvasculature (11).

DCS data were acquired at 4 Hz with an in-house built system (12, 13) consisting of a long coherence length laser operated at 852 nm (CrystaLaser, Reno, NV) and coupled to a multimode fiber (ϕ = 400 µm, NA = 0.39, FT400UMT, Thorlabs, NJ) to deliver the light to the head. Diffusely reflected light was collected using single-mode fibers (ϕ = 8.2 nm, NA = 0.14, SMF-28-J9, single-mode cutoff wavelength = 1,260 nm, Thorlabs, NJ) coupled to a photon counting module (SPCM-AQ4C, Excelitas Technologies, ON, Canada). Generated transistor-transistor logic signal (TTL) pulses were sent to an edge-detecting photon counter on a PCIe6612 counter/timer data acquisition board (National Instrument, Austin, TX; 14, 15). In-house developed software (LabVIEW, National Instrument, Austin, TX; 16) was used to record the total photon count and generate intensity autocorrelation curves consisting of 50 delay times (τ) ranging from 1 μs to 1 ms (17). The diffusively reflected light was collected at two source-detector separations (*r*_SD_) = 1 and 2.5 cm. DCS and TCD data were time-aligned within Labchart using a BNC-trigger connecting the DCS to the data acquisition software described earlier.

#### Time-resolved NIRS.

Time-resolved NIRS uses short, low-energy pulses of light (in the 650–950 nm range) paired with a time-correlated single photon counting unit to record arrival times of individual photons, which are used to build a time-of-flight distribution of diffusely reflected photons (DTOF; 18). Early arriving photons travel shorter distances, which in the context of diffusely reflected light recorded on the head, represents the light that has only traveled through superficial tissue (i.e., extracerebral). In contrast, late-arriving photons travel deeper and have a greater probability of interrogating the cerebral cortex (19). A well-established approach for obtaining depth information from recorded DTOFs is to calculate the first three statistical moments: the total number of photons (*N*), mean time of flight (*<t>*), and variance (*V*) (20, 21). Because of the right skewness of DTOFs, higher moments are more sensitive to light absorption changes in the brain, as demonstrated by Milej et al. (20, 22).

The trNIRS data were acquired at 3.33 Hz using an in-house built system (22, 23). The system was equipped with picosecond pulsed lasers operated at two wavelengths (760 and 830 nm) and a pulse repetition rate of 80 MHz (PicoQuant, Berlin, Germany). Light pulses from the two lasers’ heads were coupled into a 2.5-m long multimode bifurcated fiber (ϕ = 200 µm, NA = 0.22, Loptek, Germany). Both the emission and detection fiber bundles (ϕ = 0.75 mm, 7 × 200 µm, NA = 0.22, Loptek, Germany) were held on a subject’s forehead at *r*_SD_ = 3 cm using a 3-D-printed holder made of Flexible 80 A Resin (Formlabs Inc., MA). Diffusively reflected light collected by the detection fiber bundle was delivered to hybrid photomultiplier tubes (PMA Hybrid 50, PicoQuant, Berlin, Germany). A time-correlated single photon counting unit (HydraHarp 400, PicoQuant, Berlin, Germany) was used to record photon arrival times, and the corresponding DTOFs were built using LabView software. At the end of every study, the instrument response function (IRF) characterizing the time profile of the light source and detection system was measured using a custom-built light-tight box connecting the emission fiber to a detection probe with a separation of 9 cm. If necessary, a neutral density filter was placed in the box to avoid saturating the detector (24, 25).

### Data Analysis

To compare data obtained from different modules (TCD, DCS, NIRS, etc.), all time courses were interpolated to 1 Hz resolution. Data are presented as percent change from the 6 s average immediately before starting LBNP. Further technical details regarding the data analysis for DCS and trNIRS are described elsewhere (26).

#### Time-resolved NIRS.

Analysis of the trNIRS data is detailed in our previous works (19, 23, 26, 27). Briefly, the background signal was subtracted from each DTOF in a time series. The background signal was defined as the mean number of photons measured for the period before the initial rise of the DTOF. To determine baseline optical properties for each subject, a mean DTOF was generated from the 1-min baseline period before the first LBNP at each of the two wavelengths. The mean DTOF was fit with the solution to the diffusion equation for a semi-infinite homogeneous medium convolved with the measured instrument response function (fminsearch, MATLAB, Mathworks Inc.). The fitting parameters were μ_a0_, μ_s0_', and an amplitude factor that accounts for laser power, detection gain, and coupling efficiency.

Next, each DTOF in the time series recorded at 760 and 830 nm was analyzed to calculate the first three statistical moments: the number of photons (*N*), the mean time of flight (*<t>*), and the variance (*V*). The moments were calculated by setting the lower and upper integration limits based on arrival times corresponding to 1% of the peak of the DTOF (18). The change in each moment relative to its initial baseline value was calculated to generate three time series (i.e., Δ*N*, Δ*<t>*, and Δ*V*) for the two wavelengths individually.

To convert the time courses of changes in a statistical moment into time-varying concentrations of oxy- and deoxy hemoglobin (i.e., C_HbO_ and C_Hb_, respectively), each moment was multiplied by its corresponding sensitivity factor (19). These factors were generated separately for 760 and 830 nm using each subject’s μ_a0_(λ) and μ_s0_'(λ) values. The μ_a_(λ) time courses generated for 760 and 830 nm were converted to C_HbO_ and C_Hb_ using:

(*1*) $$\mu_{\text{a}}\left(\text{λ},t\right)=\text{ln}(10)\cdot \left({\text{ε}}_{\text{HbO}}\left(\text{λ}\right)\cdot {\text{C}}_{\text{HbO}}(t)+{\text{ε}}_{\text{Hb}}\left(\text{λ}\right)\cdot {\text{C}}_{\text{Hb}}(t)\right),$$

where ε_HbO_(λ) and ε_Hb_(λ) are the molar extinction coefficients for oxy- and deoxyhemoglobin, respectively. Changes in StO_2_ were calculated by ${\text{StO}}_{2}\left(t\right)={\text{C}}_{\text{HbO}}(t)/\left({\text{C}}_{\text{HbO}}(t)+{\text{C}}_{\text{Hb}}(t)\right)$ and $\text{HbT}\left(t\right)={\text{C}}_{\text{HbO}}(t)+{\text{C}}_{\text{Hb}}(t)$. The resulting times series were smoothed with a 3-s moving average with a zero-phase digital filter (filtfilt, MATLAB, MathWorks Inc.). The time courses of ΔStO_2_ and ΔtHb presented in the paper were derived from Δ*V*, which provides the greatest sensitivity to the brain.

#### Diffuse correlative spectroscopy.

Determination of possible changes in CBF caused by LBNP, while controlling for the confounding influence of scalp blood flow (SBF), was performed using the modified two-layer Beer–Lambert approach described by Baker et al. (28). This approach starts by measuring the sensitivity of the two source-detector distances to SBF, which was performed by applying pressure to the scalp to induce a transient reduction in SBF while acquiring intensity autocorrelation functions at the two distances. The short distance (1 cm) is primarily sensitive to extracerebral tissue, whereas the long distance (2.5 cm) contains signal contributions from both the brain and scalp. The derived scalp calibration factors were subsequently used to remove the effects of SBF on DCS data acquired during LBNP. At each time point during LBNP, the DCS measurement recorded at 1 cm was scaled by the ratio of the scalp calibration factors and subtracted from the corresponding measurement acquired at 2.5 cm. This difference signal, which represents the change in CBF at that time, was subsequently scaled by a brain sensitivity factor and normalized to an estimated baseline CBF. Performing the procedure across all time points produced a time series of relative CBF during LBNP.

Baseline CBF and the sensitivity factor for the brain were obtained using a three-layered DCS model that represents blood flow in the scalp, skull, and brain (22, 29). This analysis incorporated each individual’s optical properties measured by trNIRS (i.e., μ_a0_ and μ_s0_' at 832 nm), the coherence factor (β) determined from the average initial value of the baseline intensity autocorrelation functions, and average thicknesses of scalp (6 mm) and skull (8 mm) layers obtained from a previous study (23). Estimates of blood flow indices for scalp and brain were obtained by fitting DCS data acquired at the two source-detector distances by the three-layer model, assuming blood flow in the skull layer was negligible. The fitting was performed across all correlation times, and blood flow was modeled as a pseudo-Brownian motion (9), which has been shown to accurately track changes in CBF (23).

#### Cerebrovascular compliance and resistance.

Cerebrovascular Ci was calculated using a four-element lumped parameter modified Windkessel model. This model is described in detail elsewhere (30–32). Briefly, oscillatory flow and pressure waveforms in a vascular bed are influenced by four mechanical properties: resistance to the steady-state component of flow, as well as compliance, viscoelasticity, and inertia for the oscillatory component (31). Each of these properties can be calculated by using known mathematical relationships between the individual harmonics of corresponding flow and pressure waveforms (31). Our model prescribes and refines values of compliance, viscoelasticity, and inertia to the harmonics of the waveform until a minimal error in the model-fit is determined. For the current study, individual and corresponding brachial blood pressures and TCD waveforms were extracted for every second cardiac cycle during −80 mmHg LBNP, and input into a four-element modified Windkessel model to calculate indices of Ci. Average compliance before (“baseline”) and during (nadir) LBNP was calculated for −30 mmHg. Resistance was calculated as MAP/MCAv_mean_ for each cardiac cycle. Analysis was performed using custom written software (Matlab, Mathworks, Natick, MA).

### Statistical Analysis

Linearity and approximate normal distribution residuals were assessed qualitatively using visual inspection of Q–Q plots and formally tested with Shapiro–Wilk’s test. All variables, with exception of the ordinal Feeling Scale outcomes, met the assumptions of normality and were analyzed using a parametric approach. Friedman tests were used to assess the change in Feeling Scale responses. Student’s paired *t* tests were used to assess the effect of LBNP intensity (TCD-DCS condition) on nadir responses (percent change) during −30 mmHg and −80 mmHg. The remaining variables were assessed using one-way repeated-measures ANOVAs with a fixed factor of time (10 levels: 3 s bins across 30 s) for LBNP trials at −80 mmHg. Post hoc testing for significant interactions and main effects was completed using Bonferroni’s (parametric) multiple-comparison corrections. All results are reported using the means ± SD. Statistical analyses were conducted using Prism, version 9 (GraphPad Software Inc., San Diego).

## RESULTS

All subjects tolerated LBNP. Rapid-onset LBNP at −80 mmHg caused a small but not statistically significant decline in perceived feelings (all *P* ≥ 0.05 vs. baseline; Table 1). Low coefficients of variations (standard deviation/mean; between 60 s and 6 s baselines) confirmed that the 6 s average baseline was highly similar to the 60 s baseline recording at the beginning of each trial: MCAv_mean_ (3.1 ± 2.6%), MAP (3.2 ± 2.3%), HR (3.8 ± 2.9%), and $\text{P}{\text{ET}}_{{\text{CO}}_{2}}$ (2.4 ± 2.4%). Baseline physiological characteristics and nadir responses to rapid-onset LBNP at −30 and −80 mmHg are reported in Table 2. Rapid-onset LBNP at −80 mmHg produced significantly greater changes in MAP, HR, MCAv_mean_ pulse amplitude, and Ci compared with rapid-onset LBNP at −30 mmHg (all *P* ≤ 0.03).

**Table 1.** Perceived Feeling Scale

	Baseline	−30 mmHg LBNP	−80 mmHg LBNP
TCD-DCS condition, AU	3 ± 2	2 ± 2	2 ± 2
trNIRS condition, AU	2 ± 2	2 ± 2	1 ± 2

Feeling Scale perceptions are reported as means ± SD, based on a 11-Likert scale (−5 to +5). Nine participants (5 women) were instrumented with simultaneous diffuse correlation spectroscopy and TCD or trNIRS during two levels of rapid-onset LBNP. AU, arbitrary units; DCS, diffuse correlation spectroscopy; LBNP, lower body negative pressure; TCD, transcranial Doppler ultrasound of the middle cerebral artery; trNIRS, time-resolved near-infrared spectroscopy.

**Table 2.** Baseline and nadir values during LBNP at −30 mmHg and −80 mmHg

	Baseline	Δ% during LBNP at −30 mmHg	Δ% during LBNP at −80 mmHg	*P* Value (−30 mmHg vs. −80 mmHg)
MAP, mmHg	80 ± 10	−8 ± 7%	−22 ± 16%	**0.02**
Systolic BP, mmHg	107 ± 14	−5 ± 4%	−18 ± 16%	**0.03**
Diastolic BP, mmHg	64 ± 8	−9 ± 8%	−22 ± 16%	**0.03**
HR, beats/min	67 ± 10	4 ± 10%	21 ± 15%	**0.01**
$\text{P}{\text{ET}}_{{\text{CO}}_{2}}$, mmHg	38 ± 4	0 ± 8%	−16 ± 18%	0.05
MCAv_mean_, cm/s	65 ± 16	−1 ± 7%	−8 ± 16%	0.20
MCAv_mean_ pulse amplitude, cm/s	44 ± 8	9 ± 10%	24 ± 9%	**<0.01**
Ri, mmHg/cm/s	1.3 ± 0.4	−4 ± 5%	−14 ± 10%	**0.02**
Ci, cm/s/mmHg	5.24 ± 2.43e^−4^	17 ± 52%	322 ± 298%	**<0.01**
Microvascular CBF, cm^2^/s	2.05 ± 1.55e^−8^	1 ± 1%	−2 ± 3%	0.05

Values are means ± SD. Baseline data derived from 6 s before the start of LBNP. Nadir data are calculated as the percent change from 3 s nadir of the LBNP stimulus compared with the 6 s baseline immediately before the respective LBNP. *P* values represent statistical comparison (Student’s paired *t* test) between −30 and −80 mmHg changes. BP, blood pressure; CBF, cerebral blood flow; Ci, cerebrovascular compliance; HR, heart rate; LBNP, lower body negative pressure; MAP, mean arterial pressure; MCAv_mean_, mean blood velocity in the middle cerebral artery; $\text{P}{\text{ET}}_{{\text{CO}}_{2}}$, partial pressures of end-tidal carbon dioxide. Bolded font represents statistical significance.

### Gender Effects

No evidence of gender differences was discovered (all *P* ≥ 0.06).

### Systemic Effects

**Figure 2.**
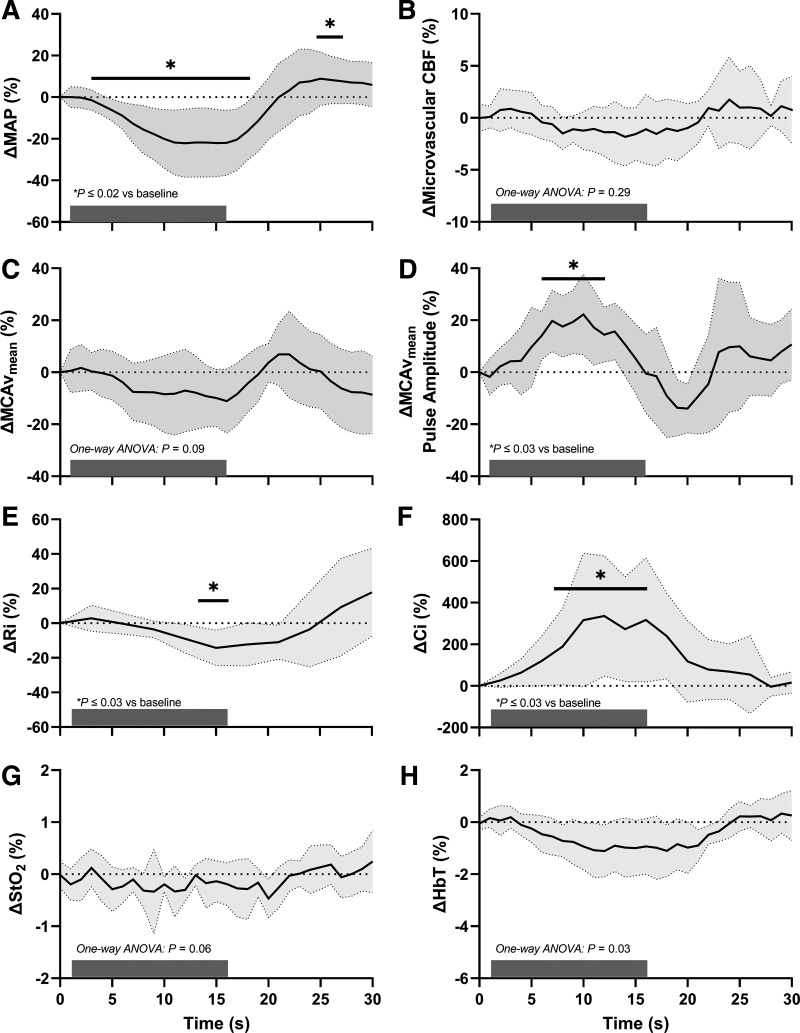
*A*–*H*: average (*n* = 9; 5 women) cerebrovascular and systemic responses to one 15 s bout of −80 mmHg rapid-onset lower body negative pressure (gray bar indicates the stimulus is on). *P ≤ 0.03 vs. baseline (timepoint 0, one-way ANOVA). CBF, cerebral blood flow; Ci, cerebrovascular compliance; HbT, total hemoglobin; MAP, mean arterial pressure; MCAv_mean_, mean blood velocity in the middle cerebral artery; Ri, resistance; StO_2_, tissue oxygen saturation.

Rapid-onset LBNP significantly reduced MAP (Table 2), with the average nadir appearing at 15 ± 2 s (Fig. 2*A*; *P* = 0.03 vs. baseline). This reduction (−22 ± 16%; Table 2) was not different than the MAP response recorded during the trNIRS condition (i.e., −20 ± 14%; *P* = 0.55 vs. TCD-DCS condition). Heart rate increased between 6 and 15 s post-onset of LBNP (all *P* ≤ 0.04 vs. baseline) and remained elevated between 18 and 24 s (both *P* ≤ 0.045 vs. baseline). $\text{P}{\text{ET}}_{{\text{CO}}_{2}}$ remained relatively constant throughout −80 mmHg rapid-onset LBNP (one-way RM ANOVA: *P* = 0.05), although it is interesting to note that the variability was driven by two individuals with an 18 and 16 mmHg reduction due to apparent sporadic breaths. As designed, the short bout of LBNP allowed us to interrogate hemodynamic responses in the absence of significant changes in cardiac output (greatest increase was 7 ± 7% at 9 s; one-way RM ANOVA: *P* = 0.19) and stroke volume (largest reduction was 12 ± 26% at 15 s; one-way RM ANOVA: *P* = 0.09).

### Cerebrovascular Effects

Both MCAv_mean_ and microvascular CBF remained relatively constant throughout rapid-onset LBNP at −80 mmHg (one-way RM ANOVA: both *P* ≥ 0.09; respectively; Fig. 2, *B* and *C*). Compared with baseline, cerebrovascular Ri was reduced during 12–15 s (*P* ≤ 0.03; Fig. 2*E*). Notably, MCAv_mean_ pulse amplitude increased from 6 to 12 s (all *P* ≤ 0.03 vs. baseline; Fig. 2*D*), driven by a significant reduction in diastolic blood velocity from 12 to 18 s (−22 ± 16% at 15 s; *P* = 0.02) in the presence of sustained systolic MCA blood velocity throughout (one-way RM ANOVA: *P* = 0.01 with nonsignificant post hoc comparisons, all *P* ≥ 0.07). This corresponds to a 20 ± 20% increase in MCA pulsatility index (*P* = 0.03 vs. baseline). Relatedly, cerebrovascular Ci increased significantly between 7 and 15 s (*P* ≤ 0.03 vs. baseline; Fig. 2*F*). StO_2_ (Fig. 2*G*) and HbT remained relatively stable throughout a single bout of −80 mmHg rapid-onset LBNP (all one-way RM ANOVA: *P* ≥ 0.06). trNIRS-derived HbT (Fig. 2*H*) tended to decrease during LBNP (one-way RM ANOVA: *P* = 0.03); however, the conservative post hoc comparisons revealed no significant changes at any point in time.

## DISCUSSION

This study utilized an emerging optical technology, DCS, combined with TCD to describe simultaneous cerebral macro- and microvascular responses to transient hypotension during a 15 s bout of LBNP. In agreement with previous studies (30, 33, 34), the results revealed that blood flow within a large cerebral artery (i.e., MCA) was preserved during a significant transient drop in MAP (i.e., 22 ± 16%). Incorporating DCS into the LBNP experiments demonstrated that CBF at the microvascular level also remained stable. This finding was supported by the trNIRS results showing no significant changes in HbT, which may be interpreted as a marker of relative changes in cerebral blood volume. The determinants of sustained flow during this period of hypotension were linked to a rapid increase in pulse amplitude and Ci of the MCA vascular bed and a subsequent dilatory response (i.e., Ri).

Like other large cerebral conduit arteries, the MCA can play a substantial role in maintaining cerebral perfusion across a range of MAP by adjusting its vessel diameter through myogenic, metabolic, and/or neural mechanisms (35–37) to alter cerebrovascular resistance. For instance, large conduit vessels are myogenically sensitive to even minor reductions in blood pressure and dilate accordingly (38). In contrast, the smallest arterioles only dilate progressively at very low levels of blood pressure (38, 39), which supports the long-standing notion that precapillary arterioles are resistance vessels and therefore express minimal elasticity (39). However, there are also passive mechanical features that contribute to cerebrovascular regulation. Large cerebral conduit arteries have high distensibility at low arterial pressures, which is attributed mainly to the stretch of elastin fibers (40). This distensibility, referred to as vascular compliance, is characterized by the vessel’s dynamic distension during systole and recoiling action during diastole and is, therefore, a fundamental property of oscillatory blood flow regulation (41). Previously, Moir et al. (30) used a Windkessel modeling approach and demonstrated large increases in cerebrovascular compliance during standing-induced hypotension, resulting in sustained systolic velocity concurrent with a reduction in diastolic velocity (30). Notably, Moir et al. (30) outlined the much earlier and transient rise in Ci compared with a delayed change in Ri. The current study supports this finding, with pulse amplitude and Ci increasing 5–6 s earlier than any significant reduction in Ri, demonstrating the primary role of passive vascular mechanisms to assist in maintaining flow as resistance mechanisms are recruited. Thus, our study provides *1*) new evidence of preserved overall perfusion of the downstream microvascular segments and *2*) further evidence of increased MCA compliance (Fig. 2*F*) and MCAv pulse amplitude (Fig. 2*D*) during periods of transient hypotension.

The Windkessel effect ensures smooth blood flow downstream from the elastic aorta (32). Herein, a vascular segment will store blood during systole and recoil to eject that flow downstream during diastole (32). Therefore, the current data suggest that the Windkessel response is sustaining microvascular flow (as illustrated in Fig. 2*B*) despite marked reductions in blood pressure. This is evident by the visual similarity between the retained levels of MCAv_mean_ (Fig. 2*C*) and DCS flow (Fig. 2*B*). The importance of this mechanism was illustrated earlier by Moir et al. (30), who demonstrated that MCAv_mean_ would be markedly reduced if cerebrovascular Ci was prevented from increasing during a sit-to-stand protocol that induced a similar orthostatic challenge. The combination of TCD and DCS is attractive for studying other conditions where there may be minimal changes in compliance, perhaps in those with increased arterial stiffness, allowing the confirmation of downstream microvascular hypoperfusion.

While speculative, we interpret the current results and those reported earlier (30) to suggest that determinants of autoregulation during transient hypotension may be different in the extracerebral conduit arteries versus the intraparenchymal microvascular segment. Specifically, both the downstream and large conduit arteries are capable of changes in resistance to flow (2). However, although all arterial segments express some degree of compliance, we propose that the changes in Ci (which reflect the entire vascular bed beyond the MCA measurement point) measured during the transient hypotension model used here are isolated primarily to the large conduit and extracerebral vessels at the base of the brain. These larger arteries provide the volumetric capacity to influence maintenance of CBF during the transient hypotensive period. If so, these results challenge the notion that changes in MCAv_mean_ represent changes in only downstream flow under the conditions of rapid blood pressure changes: rather, changes in MCA pulsatile flow patterns can also represent moments of increased local compliance (30) as flow at and beyond the compliant segment of an artery will reflect linear changes in flow through the segment but also the storage of blood volume in the large artery segment that is downstream of the TCD transducer. The combined effect of blood volume storage in compliant segments will be changes in local MCA pulsatile flow patterns during moments of increased local compliance (30), resulting in increased pulse amplitude in MCA blood velocity.

The current study combined two noninvasive technologies that are sensitive to different extremes in cerebrovascular branching to characterize the cerebral macro- and microvascular responses to transient hypotension. However, we are not the first to use these technologies during transient hypotension. To validate optical measurements of CBF with DCS, Parthasarathy et al. (8) characterized the rate of dynamic cerebral autoregulation in the MCA and microvasculature during a 14% decrease in MAP after rapid deflation of a blood pressure cuff applied to the thigh (8). In support of our findings, they report similar autoregulation rates and flow reductions between DCS measures of microvascular flow and TCD macrovascular flow. We extend these findings by highlighting the advantage of using both optical and ultrasound measurements of CBF to characterize the dynamic cerebrovascular control mechanisms, such as compliance, during transient hypotension. Furthermore, our study extends these findings to protocols with a longer, greater, and slower reduction in MAP. Thigh-cuff release stimulates an immediate and rapid drop in MAP followed by a rapid return to baseline. Indeed, Aaslid et al. (42) demonstrated that cerebral perfusion (as indexed by MCAv_mean_) was restored within ∼8 s following a rapid thigh-cuff induced 20% reduction in MAP (42). By using LBNP, the drop in MAP progressed slower than what occurs with thigh-cuff release. Nonetheless, the hypotension was sufficient to elicit changes in Ri and Ci that appeared to sustain cerebral perfusion. Additional studies are needed to determine if the speed and duration of hypotension effectively modify cerebral mechanical properties in the defense of brain perfusion. We further expand these results by providing evidence of rapid changes in MCA pulse amplitude due to increased compliance, which preserved mean flow in the early seconds of hypotension before a downstream vasoactive response that reduced vascular resistance.

### Limitations

One limitation of the present study, which was due to limited space on participants’ foreheads, was not being able to collect data simultaneously from DCS, TCD, and trNIRS. To account for variability of MAP responses to LBNP repetitions, the conditions were randomized. A second limitation was the DCS modeling analysis relied on assuming the distance from the probe to the brain (e.g., scalp and skull thicknesses). We have clarified the effect of these assumptions on DCS measures of flow in Shoemaker et al.(26). Although it may be possible to include the overall thickness as an additional fitting variable (28), we elected to use measured values from a previous study (23) since the probe location on the forehead and demographics of the participants were similar between the two studies (i.e., young, healthy adults). Furthermore, we were unable to assess compliance in the microvasculature due to inadequate cerebral pulsatile signal quality, which is required by our Windkessel modeling approach. A fourth consideration is the inability to measure blood pressure in the brain, which is a common limitation when assessing the cerebral circulation with noninvasive technologies. Therefore, we likely are overestimating the total reduction in microcirculatory pressure by assuming MAP. Last, the current findings are derived from a small sample size and delimited to only young, healthy individuals and not aging or clinical populations.

### Conclusions

The current study utilized rapid-onset LBNP to explore the hemodynamic responses of both the cerebral macro- and microvascular beds during a period of transient hypotension. This study produced the main finding that rapid increases in compliance during transient hypotension support a continuance of cerebral blood flow at the microcirculatory level. Specifically, our data revealed a rapid increase in cerebrovascular compliance during 15 s of rapid-onset LBNP with marked hypotension, with no significant change in macro- or microvascular CBF. The data are supported by no significant change in NIRS-derived measures or StO_2_ or HbT. Thus, this study demonstrates the utility of simultaneous, depth-enhanced optical and ultrasound measures of CBF to assess the cooperative and dynamic features of cerebrovascular regulation.

## DATA AVAILABILITY

The data that support the findings of this study are available from the corresponding author upon reasonable request. 

## GRANTS

This work was funded through grants from the Canadian Institutes of Health Research (Grant No. 130391) and the Natural Sciences and Engineering Research Council of Canada (Grant No. R3592A02002 to K.S.L. and Grant No. RGPIN-2018-06255 to J.K.S.).

## DISCLOSURES

L.S. and J.K.S. are relatives. None of the other authors have any conflicts of interest, financial or otherwise, to disclose.

## AUTHOR CONTRIBUTIONS

L.N.S., D.M., K.S.L., and J.K.S. conceived and designed research; L.N.S., D.M., A.S., and J.M. performed experiments; L.N.S., D.M., A.S., and J.M. analyzed data; L.N.S., D.M., K.S.L., and J.K.S. interpreted results of experiments; L.N.S. prepared figures; L.N.S. drafted manuscript; L.N.S., D.M., A.S., J.M., K.S.L., and J.K.S. edited and revised manuscript; L.N.S., D.M., A.S., J.M., K.S.L., and J.K.S. approved final version of manuscript. 
